# Clarifying the Use of Aggregated Exposures in Multilevel Models: Self-Included vs. Self-Excluded Measures

**DOI:** 10.1371/journal.pone.0051717

**Published:** 2012-12-10

**Authors:** Etsuji Suzuki, Eiji Yamamoto, Soshi Takao, Ichiro Kawachi, S. V. Subramanian

**Affiliations:** 1 Department of Epidemiology, Okayama University Graduate School of Medicine, Dentistry and Pharmaceutical Sciences, Okayama, Japan; 2 Department of Information Science, Faculty of Informatics, Okayama University of Science, Okayama, Japan; 3 Department of Society, Human Development, and Health, Harvard School of Public Health, Boston, Massachusetts, United States of America; Zhongshan Ophthalmic Center, China

## Abstract

**Background:**

Multilevel analyses are ideally suited to assess the effects of ecological (higher level) and individual (lower level) exposure variables simultaneously. In applying such analyses to measures of ecologies in epidemiological studies, individual variables are usually aggregated into the higher level unit. Typically, the aggregated measure includes responses of every individual belonging to that group (i.e. it constitutes a self-included measure). More recently, researchers have developed an aggregate measure which excludes the response of the individual to whom the aggregate measure is linked (i.e. a self-excluded measure). In this study, we clarify the substantive and technical properties of these two measures when they are used as exposures in multilevel models.

**Methods:**

Although the differences between the two aggregated measures are mathematically subtle, distinguishing between them is important in terms of the specific scientific questions to be addressed. We then show how these measures can be used in two distinct types of multilevel models—self-included model and self-excluded model—and interpret the parameters in each model by imposing hypothetical interventions. The concept is tested on empirical data of workplace social capital and employees' systolic blood pressure.

**Results:**

Researchers assume group-level interventions when using a self-included model, and individual-level interventions when using a self-excluded model. Analytical re-parameterizations of these two models highlight their differences in parameter interpretation. Cluster-mean centered self-included models enable researchers to decompose the collective effect into its within- and between-group components. The benefit of cluster-mean centering procedure is further discussed in terms of hypothetical interventions.

**Conclusions:**

When investigating the potential roles of aggregated variables, researchers should carefully explore which type of model—self-included or self-excluded—is suitable for a given situation, particularly when group sizes are relatively small.

## Introduction

An attractive feature of multilevel analyses is their ability to assess the effects of ecological (higher level) and individual (lower level) exposure variables simultaneously [1]–[8]. In the majority of applications involving measures of ecologies in epidemiological studies, individual variables are aggregated into the higher level unit [4], [6]. This approach is commonplace in studies on the association between social trust and health, in which social trust at a high level (e.g. neighborhood, workplace or school) is defined by aggregating the responses of individuals of the clusters [8]–[10]. Typically, the aggregated measure includes responses of all individuals belonging to that group, which we designate the “self-included measure.” More recently, researchers have developed an aggregate measure that excludes the response of the individual to whom the aggregate measure is linked [11], [12], which we term the “self-excluded measure.” It is important to note that the two are distinctly different measures. The implicit motivation for using the self-excluded measure has been concerns regarding how to assess the effect of a reduced form of aggregated social capital on individual-level outcome, while (at least) mitigating potential bias arising from omitted variables.

In this study, we clarify the substantive and technical properties of the two types of aggregated measures when they are used as exposures in multilevel models. Both measures can be used to assess certain ecologic effects, or group effects of exposure (e.g. social capital) on the individual-level outcome. Although the differences between the measures are mathematically subtle, distinguishing between them has implications related to the specific scientific questions to be addressed. In this way, plausible distinct causal interpretation of the estimated coefficients in multilevel models may be achieved.

## Methods

### Self-included and self-excluded measures

Let *x_ij_* denote an individual-level exposure representing workplace social capital score of individual *i* in group *j* (e.g. work unit, division, company). We also let *n_j_* denote the size of group *j*. Social capital at the individual level refers to the individual's perceptions of social cohesion in the workplace (e.g. their personal assessments of whether their co-workers are helpful and trustworthy). When these individual perceptions are aggregated at the level of the workplace, they constitute group-level social capital, i.e. the average level of trustworthiness or helpfulness, as perceived by the workers. Then, as briefly explained in the Introduction, two types of aggregated social capital scores—self-included measure and self-excluded measure—can be calculated according to the groups.

Self-included measure is the arithmetic mean of social capital scores of all individuals in the group *j*, calculated as 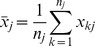 (Table 1). From the substantive perspective, the self-included measure is used primarily to measure social capital as a group-level attribute, assessing its “proxy” by aggregating social capital scores, including the person being observed [13]. Thus, when researchers employ the self-included measure, the specific scientific question of interest becomes the relationship between the group-level attribute and the individual-level health outcome.

**Table 1:** Example of a hypothetical data set.

Level-1 id	Level-2 id	Individual responses	Self-included measure	Self-excluded measure
1	1	7	5.50	5.00
2	1	4	5.50	6.00
3	1	5	5.50	5.67
4	1	6	5.50	5.33
5	2	4	4.60	4.75
6	2	3	4.60	5.00
7	2	3	4.60	5.00
8	2	8	4.60	3.75
9	2	5	4.60	4.50
10	3	3	6.75	8.00
11	3	7	6.75	6.67
12	3	9	6.75	6.00
13	3	8	6.75	6.33
:	:	:	:	:

By contrast, self-excluded measure is an aggregated (group-level) social capital at work, excluding the individual being observed. Thus, the self-excluded measure is the arithmetic mean of individual coworkers' responses from the same group *j*, described as 
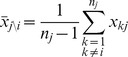
. Notably, the self-excluded measure can take different values between workers nested within the *same* group (Table 1). Substantively, the self-excluded measure assesses the social capital defined in terms of resources embedded in social networks around an individual [13]. Now, the scientific question of interest becomes the relationship between resources available to oneself and his/her health outcome. Note that both self-included and self-excluded measures are independent of the network topology.

In the next section, we show how these measures can be used in two distinct types of multilevel models—self-included model and self-excluded model—and interpret the parameters in each model by imposing hypothetical interventions.

### Self-included and self-excluded models

The archetypal self-included multilevel model would base group-level social trust on employee response clusters, and calculate the mean of all workers' scores (i.e. would involve a self-included measure). Such a self-included model can be written as follows:
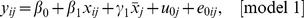
where *y_ij_* is an observed outcome of individual *i* in group *j*. Group-level random effect of the intercept (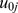) is assumed to be normally distributed with a mean of 0 and variance 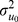, whereas individual-level random error of the intercept (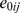) is assumed normally distributed with a mean of 0 and variance 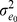. To enhance the readability of the statistical models, we omit the covariates throughout the manuscript. The parameter *γ*_1_ is the expected change in the individual-level outcome when the group-level social capital (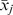) is increased by 1 unit (holding all other components of the model constant). This may however pose a challenge in the interpretation of *γ*_1_ since, if *x_ij_* is maintained constant, a unit change in 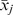 corresponds to a very specific change in the mean for the remaining individuals in the group. Furthermore, the classic formulation of model 1 is susceptible to high collinearity between the individual- and group-level exposures of social capital, leading to poor precision [2], [5], [14].

One solution is to reformulate model 1 with *x_ij_* centered on its cluster mean (i.e. group-level social capital) [2], [5], [14]. The reformulated model is expressed as:
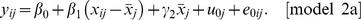



Model 2a is simply a re-parameterization of model 1, in which the coefficient of the aggregated social capital (*γ*_1_) of model 1 has been replaced by (*γ*_2_–*β*_1_). However, in model 2a, the individual-level social capital variable, 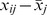, is orthogonal to its group-level counterpart 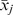, thus overcoming the collinearity problem that persists in model 1 (see Text S1) [2], [5].

Centering the individual-level social capital on its cluster-mean is doubly beneficial. First, it allows us to envisage the hypothetical intervention when using the self-included measure. Recall that in this case researchers are interested in measuring social capital as a group-level attribute. Thus, the parameter of primary interest is *γ*_2_, which represents the expected change in the individual-level outcome of individual *i* in group *j*, when the group-level social capital (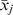) is increased by 1 unit and other components of the model are held constant. Notably, this requires that social capital score of individual *i* also increases by 1 unit so that the subtraction, 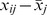, is unchanged for that individual. In other words, the individual in question (i.e. individual *i*) is an average person in terms of “susceptibility” to the hypothetical group-level intervention. Note that although the unit of randomization is the group itself, particular interventions could be imposed at either the individual or at the group level. Previously, potential psychosocial and health effects of organizational-level interventions have been examined in occupational settings [15], [16]. An alternative type of group-level intervention focuses on the parameter *β*_1_. In this case, researchers assume a constant group-level social capital score, and instead change the “relative placement” of (two or more) individuals in the corresponding group, such that the social capital score of individual *i* increases by 1 unit while the group-level social capital score of group *j* remains constant. The output is again the change in individual-level outcome. Note that, although the aggregated measure is retained constant, hypothetical intervention is administered at the group level; interventions are randomly assigned to groups. Regardless of whether the parameter of interest is *γ*_2_ or *β*_1_, model 2a assumes group-level interventions.

The second benefit of cluster-mean centering procedure is its ability to disentangle the within- and between-group components of the social capital measure: *β*_1_ measures the pure “individual effect” of social capital on the individual-level outcome *within* a group [2], [5]. Such an effect is termed a “within-cluster effect” [2]. By contrast, *γ*_2_ measures the aggregate effect of (group-level) social capital on individual-level outcomes between groups, which constitutes a “collective effect” because the samples are aggregates of characteristics associated with all individuals in the corresponding group [17], [18]. This effect has been also called “between-cluster effect” in some literatures [2]. Finally, the extent to which the magnitude of between-cluster relationship (*γ*_2_) differs from the within-cluster effect (*β*_1_) has been called “compositional effect” [2], simply equal to (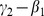). Although this effect is based on characteristics specific to the individuals in particular clusters, individual characteristics are intrinsic components of the cluster since they are putatively distributed non-randomly across clusters [17], [18]. We emphasize that compositional explanations may differ from individual explanations, because differential cluster composition may arise from extra-individual processes and need not reflect individual choice [17], [18]. Note also that collective effect can be decomposed into individual and compositional effects. In other words, a nonzero estimate for *γ*_2_ does not necessarily imply a compositional effect; if *β*_1_ and *γ*_2_ are equal, no compositional effect is present [2]. Models 1 and 2a are equivalent, although model 2a is superior in that its level-2 aggregate measure is uncorrelated with its analogous level-1 measure. Thus, in this paper, the self-included model will be based on model 2a. (Models that incorporate a cluster-mean exposure variable have been also termed “hybrid fixed effect models” [19].).

By contrast, in a self-excluded model, the self-excluded measures are linked to each member of the group. The self-excluded model can be written as follows:
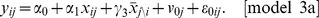



Group-level random effect of the intercept (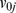) is assumed to be normally distributed with a mean of 0 and variance 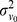, whereas individual-level random error of the intercept (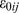) is assumed normally distributed with a mean of 0 and variance 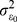. As explained in the previous section, the “group-level” social capital in model 3a can take different values between workers nested within the *same* group. In other words, the group-level social capital is defined not as an ecological variable (at level 2) but as an individual-level variable (at level 1) in model 3a. Recall that the self-excluded measure is used to describe social capital in terms of resources embedded in social networks surrounding an individual. Accordingly, the parameter of primary interest is *γ*_3_, which represents the expected change in the individual-level outcome of individual *i* in group *j*, when the mean of the individual's co-workers social capital scores (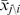) is increased by 1 unit (holding all other components of the model constant). This type of individual-level intervention may be properly realized when a specific individual is randomly moved to another group, e.g. personnel relocation. In this case, the environment of individual *i* changes while his/her social capital score does not. When using the self-excluded model, however, researchers could envisage an alternative individual-level intervention by focusing on the parameter 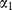. For instance, one might be interested in likely changes in individual-level outcome if the social capital score of the individual is altered without group change. In this case the situation reverses; the social capital score of individual *i* changes while his/her “environment” remains static. In other words, the hypothetical intervention is assumed to influence only the specific individual. Regardless of whether the parameter of interest is *γ*_3_ or 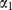, individual-level intervention is assumed in model 3a. It is notable that, unlike model 2a, the collinearity problem cannot be completely overcome in model 3a (see Text S1).

Table 2 summarizes the parameter interpretations in models 2a and 3a, highlighting their differences in terms of hypothetical interventions. In the cluster-mean centered self-included model, researchers assume group-level intervention, whereas in the self-excluded model, they assume individual-level intervention. Both types of studies have been used in a mutually complementary manner in studies of neighborhood effects [20]. Recently, Suzuki [21] presented an analogous discussion in the context of “temporal dimension”.

**Table 2:** Interpretations of parameters in cluster-mean centered self-included model and self-excluded model.

Model a	Parameter	Interpretations based on hypothetical interventions
Cluster-mean centered self-included model (model 2a)		
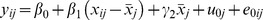	*β*_1_	(group-level intervention) Expected change in the individual-level outcome of individual *i* of group *j*, following changes in “relative placement” of individuals in the group, such that the individual-level measure of individual *i* increases by 1 unit while the self-included measure of group *j* remains constant.
	*γ*_2_	(group-level intervention) Expected change in the individual-level outcome of individual *i* of group *j*, following an intervention such that the self-included measure of group *j*, and the individual-level measure of individual *i* both increase by 1 unit.
Self-excluded model (model 3a)		
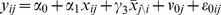	*α*_1_	(individual-level intervention) Expected change in the individual-level outcome of individual *i* of group *j*, following an exclusive increase in individual-level measure of individual *i* by 1 unit, while individual *i* remains in group *j* and the self-excluded measure of group *j* remains constant.
	*γ*_3_	(individual-level intervention) Expected change in the individual-level outcome of individual *i* of group *j*, following a shift of individual *i* to another group, such that the self-excluded measure of group *j* increases by 1 unit while the individual-level measure of individual *i* remains constant.

a As explained in the main text, *y_ij_* is an observed outcome of individual *i* in group *j* and *x_ij_* is an individual-level social capital score of individual *i* in group *j*. Furthermore, 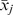 is a self-included measure that denotes the mean of social capital scores of all individuals in group *j*. It is calculated as 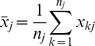, where *n_j_* is the size of group *j*. Similarly, 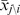 is a self-excluded measure denoting the mean of social capital scores of all individuals excepting individual *i* in group *j*, calculated as 
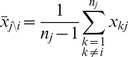
.

## Results

### Re-parameterization of models

In this section, we show how both self-included and self-excluded models can be re-parameterized. These re-parameterizations are not intended to show the relations between these two models; rather they highlight the subtle interpretation differences between the models for each parameter. When showing the results of re-parameterizations, it is desirable to attribute meaningful terminology to the parameters in model 3a. To this end, we tentatively assign the term “self effect” to the coefficient of individual-level social capital (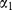), and the term “others effect” to the coefficient of the self-excluded measure (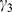). In sociological literature, these two effects may be called “ego effect” and “alters effect,” respectively, concordant with network analysis terminologies [22], [23]. The summation of the two effects is termed “all effect” in this paper.

The cluster-mean centered self-included model (model 2a) can be rewritten as follows (see Table 3):

**Table 3:** Decomposition of effect of cluster-mean centered self-included model (2a) and its re-parameterized form (2b).

Model a	Collective effect b	Decomposition of collective effect
Original model (model 2a)		
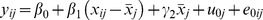	*γ*_2_	Individual effect c: *β_1_*
		Compositional effect: *γ*_2−_ *β*_1_
Re-parameterized model (model 2b)		
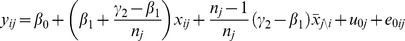	*γ*_2_	Self-like effect: 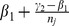
		Others-like effect: 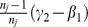

a As explained in the main text, *y_ij_* is an observed outcome of individual *i* in group *j*, *x_ij_* is an individual-level social capital score of individual *i* in group *j*, 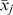 = 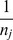 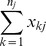 is the mean of social capital scores of all individuals in group *j*, *n_j_* is the size of the group *j*, and 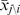 =  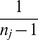 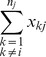 is the mean of social capital scores of all individuals (excluding individual *i*) in group *j*.

b This effect is also known as between-cluster effect.

c This effect is also known as within-cluster effect.



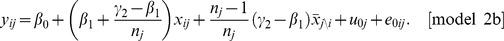


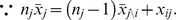



Because the coefficients of both individual-level social capital (*x_ij_*) and (self-excluded) group-level social capital (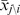) in model 2b are functions of the size of a given group *j* (*n_j_*), the individual workers nested within group *j* should have identical coefficient estimates, although these can vary across groups. In other words, we cannot obtain “constant” estimates for all individuals across groups in model 2b; rather, cluster-specific estimates can be obtained by specifying the size of the cluster of interest and inserting an appropriate *n_j_*. In model 2b, the coefficient of individual-level social capital may be interpreted as a “self-like effect” whereas the coefficient of self-excluded aggregated variable represents an “others-like effect.” As shown in Table 3, model 2b provides a subtly different decomposition of the collective effect in model 2a; the self-like effect in model 2b exceeds the individual effect in model 2a by 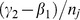, whereas the others-like effect in model 2b is smaller than the compositional effect in model 2a by the same quantity. This quantity may therefore be interpreted as an individual's average contribution to compositional effect (or “extra-individual” effect) in model 2a.

In the same manner, the self-excluded model (model 3a) can be rewritten as (see Table 4):

**Table 4:** Decomposition of effect of self-excluded model (3a) and its re-parameterized form (3b).

Model a	All effect	Decomposition of all effect
Original model (model 3a)		
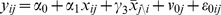	*α*_1_+*γ*_3_	Self effect: *α*_1_
		Others effect: *γ*_3_
Re-parameterized model (model 3b)		
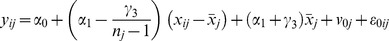	*α*_1_+*γ*_3_	Individual-like effect: 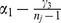
		Compositional-like effect: 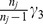

a As explained in the main text, *y_ij_* is an observed outcome of individual *i* in group *j*, *x_ij_* is an individual-level social capital score of individual *i* in group *j*, 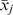 = 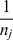 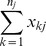 is the mean of social capital scores of all individuals in group *j*, *n_j_* is the size of the group *j*, and 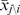 = 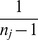 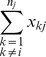 is the mean of social capital scores of all individuals (excluding individual *i*) in group *j*.



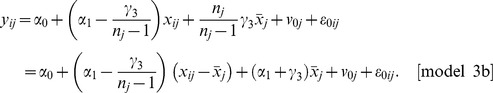


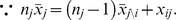



The coefficients of cluster-mean centered individual-level social capital variables (*x_ij_*–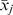) are again functions of the *j*th group size (*n_j_*). This implies that the coefficient estimate of individual workers nested within the same group is identical, though it can vary across groups. Notably, however, the coefficient of group-level social capital (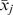) is not a function of *n_j_*, indicating that its estimate is identical for all groups. In model 3b, the coefficient of cluster-mean centered individual-level social capital constitutes an “individual-like effect” whereas the coefficient of a self-included aggregated variable is an “all effect.” Thus, as shown in Table 4, model 3b provides a subtly different decomposition of the all effect in model 3a; the individual-like effect in model 3b is smaller than the self effect in model 3a by 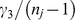, whereas the compositional-like effect in model 3b exceeds the others effect in model 3a by the same quantity.

### Illustration

#### Ethics Statement

This study on Epidemiological Research was reviewed and approved by the Ethics Committee of the Okayama University Graduate School of Medicine, Dentistry and Pharmaceutical Sciences.

#### Data Set

We apply the model to empirical data on workplace social capital and employees' systolic blood pressure (SBP). The workplace is a manufacturing company in Shizuoka prefecture, Japan. Data are derived from an annual health checkup and questionnaires administered between May and October 2009. Of the 1664 study subjects, 1601 participants returned the questionnaire (response rate: 96.2%, 1314 men and 287 women). We excluded 5 subjects whose work unit was not identified. Consistent with previous studies adopting the self-excluded measure [11], [12], a further 6 subjects were excluded because they worked in units containing less than 3 employees. Finally, we excluded respondents who did not fully complete the social capital questions or record their SBP. As a result, 1077 workers, nested within 95 work units, were ultimately eligible for analysis. The median work unit size was 10 employees (interquartile range: 6–20; range: 3–89).

Workplace social capital was based on 20 responses, each obtained on a 5-point Likert scale (strongly agree, somewhat agree, neither agree nor disagree, somewhat disagree, and strongly disagree; see Text S2). The individual-level social capital was the sum of response scores (range 0–80) with higher score indicating a higher social workplace capital. The mean of individual-level social capital score was 45.8. We calculated both self-included and self-excluded measures as outlined above.

The fixed and random parameter estimates (along with their standard errors) for a multilevel linear regression were obtained using MLwiN 2.22 [24]. The current example is for illustrative purposes only, and is adjusted for sex and age (continuous) as covariates to simplify the discussion.

#### Results

Table 5 shows the results of cluster-mean centered self-included model and self-excluded model. In the former model, the coefficient of individual-level social capital (i.e. individual effect) is 0.042 mmHg. This is the expected change in SBP of individual *i* in work unit *j*, when the placement of the individual is changed such that the social capital score of the individual increases by 1 unit while the work unit-level social capital of unit *j* is maintained constant. Likewise, the coefficient of work unit-level social capital (i.e. collective effect) is 0.136 mmHg. This quantity is the expected change in SBP of individual *i* in work unit *j*, if both the work unit-level social capital of group *j*, and the individual-level social capital of individual *i*, increase by 1 unit (in the self-included model, a work unit-level intervention is implemented). The compositional effect is then 0.094 mmHg (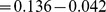). By contrast, in the self-excluded model, the coefficient of individual-level social capital (i.e. self effect) is 0.047 mmHg, representing the expected change in SBP of individual *i* in work unit *j*, when the social capital score of individual *i* is increased by 1 unit while the work unit-level social capital of group *j* does not change. Finally, the coefficient of work unit-level social capital (i.e. others effect) was 0.091 mmHg, representing the expected change in SBP of individual *i* in work unit *j*, when individual *i* moves to another group, such that the self-excluded measure increases by 1 unit while the social capital score of individual *i* does not change (in the self-excluded model, an individual-level intervention is implemented).

**Table 5:** Effects of individual-level and work unit-level social capital on systolic blood pressure of workers, Japan, 2009.

		Cluster-mean centered self-included model		Self-excluded model	
		Estimate	(95% CI)	Estimate	(95% CI)
Fixed	Intercept	120.050	(109.125, 130.975)	119.959	(109.336, 130.582)
*Individual-level variables*	Individual-level social capital a	0.042	(−0.048, 0.132)	0.047	(−0.039, 0.133)
	Women (vs. men)	−5.286	(−8.510, −2.062)	−5.283	(−8.505, −2.061)
	Age (year) b	0.532	(0.430, 0.634)	0.532	(0.430, 0.634)
*Work unit-level variable*	Work unit-level social capital c	0.136	(−0.101, 0.373)	0.091	(−0.138, 0.320)
Random	Individual-level variance (SE)	314.193	(13.852)	314.176	(13.851)
	Work unit-level variance (SE)	1.791	(3.311)	1.789	(3.309)

CI, confidence interval; SE, standard error.

a The individual-level social capital was assessed on a scale ranging from 0 to 80, with higher values indicating higher social capital.

b Age was grand-mean centered.

c Work unit-level social capital was defined as the mean of all workers' scores in the work unit in the self-included model, whereas it was defined as the mean of coworkers' responses in the self-excluded model.

Table 6 shows the results of cluster-mean centered self-included model and its re-parameterization, for the 10 smallest and the 10 largest work units (results were computed for all of the work units, but we display only a small subset to illustrate the model's performance). Values of the two effects that are derived from the re-parameterized model vary with size of work unit (*n_j_*). As the size of the work unit increases further, however, the effects from the original and re-parameterized models, by definition, converge. Indeed, for cluster size exceeding 10, both effects are essentially the same. Equivalent results for the self-excluded model and its re-parameterization are displayed in Table 7.

**Table 6:** Numerical outputs of cluster-mean centered self-included model and its re-parameterized form (as functions of the work unit size *n_j_*).

	Original model		Re-parameterized model	
*n_j_*	individual effect	compositional effect	self-like effect	others-like effect
3	0.042	0.094	0.073	0.063
4	0.042	0.094	0.066	0.071
5	0.042	0.094	0.061	0.075
6	0.042	0.094	0.058	0.078
7	0.042	0.094	0.055	0.081
8	0.042	0.094	0.054	0.082
9	0.042	0.094	0.052	0.084
10	0.042	0.094	0.051	0.085
11	0.042	0.094	0.051	0.085
12	0.042	0.094	0.050	0.086
45	0.042	0.094	0.044	0.092
49	0.042	0.094	0.044	0.092
52	0.042	0.094	0.044	0.092
56	0.042	0.094	0.044	0.092
58	0.042	0.094	0.044	0.092
62	0.042	0.094	0.044	0.092
72	0.042	0.094	0.043	0.093
77	0.042	0.094	0.043	0.093
84	0.042	0.094	0.043	0.093
89	0.042	0.094	0.043	0.093

Results are shown for the 10 smallest and 10 largest work units only.

**Table 7:** Numerical outputs of self-excluded model and its re-parameterized form (as functions of the work unit size *n_j_*).

	Original model		Re-parameterized model	
*n_j_*	self effect	others effect	individual-like effect	compositional-like effect
3	0.047	0.091	0.002	0.137
4	0.047	0.091	0.017	0.121
5	0.047	0.091	0.024	0.114
6	0.047	0.091	0.029	0.109
7	0.047	0.091	0.032	0.106
8	0.047	0.091	0.034	0.104
9	0.047	0.091	0.036	0.102
10	0.047	0.091	0.037	0.101
11	0.047	0.091	0.038	0.100
12	0.047	0.091	0.039	0.099
45	0.047	0.091	0.045	0.093
49	0.047	0.091	0.045	0.093
52	0.047	0.091	0.045	0.093
56	0.047	0.091	0.045	0.093
58	0.047	0.091	0.045	0.093
62	0.047	0.091	0.046	0.092
72	0.047	0.091	0.046	0.092
77	0.047	0.091	0.046	0.092
84	0.047	0.091	0.046	0.092
89	0.047	0.091	0.046	0.092

Results are shown for the 10 smallest and the 10 largest work units only.

## Discussion

In this study, we clarified the substantive and technical properties of two distinct types of aggregated measures–self-included and self-excluded measures. The former is identical among individuals nested within the same group, whereas the latter can, by definition, take different values between individuals nested within the same group. In other words, although the self-excluded measure is an aggregated measure, it is not an ecological variable but a level-1 variable. We then adopted these measures in their respective distinct multilevel models (self-included and self-excluded). Although the mathematical differences between the two models are not large, they are substantively and analytically significant. We highlighted these distinctions by implementing hypothetical interventions, assuming group-level interventions for the self-included model, and individual-level interventions for the self-excluded model.

With regard to multilevel models in general, it has been frequently argued that the individual-level coefficient of primary interest is the pooled-within-organization relationship [2]. In other words, the estimated coefficients of multilevel models may be interpreted similarly to those of stratified analysis and ordinary regression [8]. Consequently, cluster-mean centered self-included models are effective tools by which to decompose the collective effect into its within- and between-group components. Indeed, the cluster-mean centering procedure is viable even when the exposure of interest is a dichotomous variable [2], as demonstrated in recent studies on workplace social capital [25], [26]. That the cluster-mean centering procedure resolves collinearity is almost common knowledge; here, we aimed to show another significant benefit when interpreting the estimated coefficients by imposing hypothetical intervention on cluster-mean centered multilevel models. Indeed, as interest in the potential group-level determinants of health has recently surged [1], [15], [16], [27], [28], an enhanced understanding of multilevel models, which would benefit health studies to no small extent, is timely. On a related theme, other forms of network models (e.g. exponential random graph models), which have been utilized in social science studies to analyze complex network data [29]–[31], have also been recently adopted in biological networks [32], [33].

From an analytical perspective, re-parameterizations of self-included and self-excluded models could assist researchers in interpreting the model parameters. Overall, self-excluded models place more emphasis on individuals than groups. Although the effects from original and re-parameterized models converge as group size increases, researchers should carefully identify which models are appropriate to a situation, because the different models lead to different interpretations of the estimated coefficients.

As noted previously [4], [6], various researchers have categorized ecological variables in different ways. Furthermore, no consistent definitions have been assigned to the effects estimated from multilevel models. In particular, compositional effect has been confused with individual effect, while compositional and contextual explanations have been largely regarded as mutually exclusive and competing, as noted previously [18], [34], [35]. In the present paper, we defined compositional effect as an “extra-individual” effect; that is, it derives from individual characteristics in the corresponding group [2]. Although this paper is not intended to thoroughly review and clarify the terminologies in multilevel analysis, we emphasize a need for further studies to give consistent definitions as well as to correctly interpret each effect.

In conclusion, this study has clarified the use of aggregated exposures in multilevel models, focusing on self-included and self-excluded measures. The distinctions between these two models are especially relevant to social science research, including studies on social capital. By imposing hypothetical interventions, we showed that the cluster-mean centered self-included model is useful for exploring the effects of group-level interventions, whereas a self-excluded model is suitable for exploring the effects of individual-level interventions. In particular, the differences between the models are amplified for small group size (group size ≤10). Since the scientific questions addressed by researchers are distinct it is critical that an appropriate model be used in a given situation. Future studies could be enriched by investigating the potential roles of aggregated variables, clarifying their meaning and employing an appropriate analytical procedure.

## Supporting Information

Text S1 **Sample covariance between individual- and group-level social capital variables in models 1, 2a, and 3a.**
(PDF)

Text S2 **Items used to measure workplace social capital.**
(PDF)

## References

[1] Diez-Roux AV (2000) Multilevel analysis in public health research. Annu Rev Public Health 21: 171–192. 10884951 10.1146/annurev.publhealth.21.1.171

[2] Raudenbush SW, Bryk AS (2002) Hierarchical Linear Models: Applications and Data Analysis Methods. Thousand Oaks, CA: Sage Publications.

[3] Subramanian SV, Jones K, Duncan C (2003) Multilevel methods for public health research. In: Kawachi I, Berkman LF, editors. Neighborhoods and Health. New York, NY: Oxford University Press. pp. 65–111.

[4] Blakely T, Subramanian SV (2006) Multilevel studies. In: Oakes JM, Kaufman JS, editors. Methods in Social Epidemiology. San Francisco, CA: Jossey-Bass. pp. 316–340.

[5] Bingenheimer JB, Raudenbush SW (2004) Statistical and substantive inferences in public health: issues in the application of multilevel models. Annu Rev Public Health 25: 53–77. 15015912 10.1146/annurev.publhealth.25.050503.153925

[6] Blakely TA, Woodward AJ (2000) Ecological effects in multi-level studies. J Epidemiol Community Health 54: 367–374. 10814658 10.1136/jech.54.5.367 PMC1731678

[7] Duncan C, Jones K, Moon G (1998) Context, composition and heterogeneity: using multilevel models in health research. Soc Sci Med 46: 97–117. 9464672 10.1016/s0277-9536(97)00148-2

[8] Kaufman JS (2008) Social epidemiology. In: Rothman KJ, Greenland S, Lash TL, editors. Modern Epidemiology. 3rd ed. Philadelphia, PA: Wolters Kluwer Health/Lippincott Williams & Wilkins. pp. 532–548.

[9] Kawachi I, Subramanian SV, Kim D (2008) Social capital and health: A decade of progress and beyond. In: Kawachi I, Subramanian SV, Kim D, editors. Social Capital and Health. New York, NY: Springer. pp. 1–26.

[10] Subramanian SV, Lochner KA, Kawachi I (2003) Neighborhood differences in social capital: a compositional artifact or a contextual construct? Health Place 9: 33–44. 12609471 10.1016/s1353-8292(02)00028-x

[11] Kouvonen A, Oksanen T, Vahtera J, Stafford M, Wilkinson R, et al (2008) Low workplace social capital as a predictor of depression: the Finnish Public Sector Study. Am J Epidemiol 167: 1143–1151. 18413361 10.1093/aje/kwn067

[12] Kouvonen A, Oksanen T, Vahtera J, Väänänen A, De Vogli R, et al (2008) Work-place social capital and smoking cessation: the Finnish Public Sector Study. Addiction 103: 1857–1865. 18705683 10.1111/j.1360-0443.2008.02315.x

[13] Kawachi I (2006) Commentary: social capital and health: making the connections one step at a time. Int J Epidemiol 35: 989–993. 16870679 10.1093/ije/dyl117

[14] Enders CK, Tofighi D (2007) Centering predictor variables in cross-sectional multilevel models: a new look at an old issue. Psychol Methods 12: 121–138. 17563168 10.1037/1082-989X.12.2.121

[15] Egan M, Bambra C, Thomas S, Petticrew M, Whitehead M, et al (2007) The psychosocial and health effects of workplace reorganisation. 1. A systematic review of organisational-level interventions that aim to increase employee control. J Epidemiol Community Health 61: 945–954. 17933951 10.1136/jech.2006.054965 PMC2465601

[16] Bambra C, Egan M, Thomas S, Petticrew M, Whitehead M (2007) The psychosocial and health effects of workplace reorganisation. 2. A systematic review of task restructuring interventions. J Epidemiol Community Health 61: 1028–1037. 18000123 10.1136/jech.2006.054999 PMC2465678

[17] Subramanian SV, Kubzansky L, Berkman LF, Fay M, Kawachi I (2006) Neighborhood effects on the self-rated health of elders: uncovering the relative importance of structural and service-related neighborhood environments. J Gerontol B Psychol Sci Soc Sci 61: S153–160. 16670193 10.1093/geronb/61.3.s153

[18] Macintyre S, Ellaway A, Cummins S (2002) Place effects on health: how can we conceptualise, operationalise and measure them? Soc Sci Med 55: 125–139. 12137182 10.1016/s0277-9536(01)00214-3

[19] Schempf AH, Kaufman JS (2012) Accounting for context in studies of health inequalities: a review and comparison of analytic approaches. Ann Epidemiol 22: 683–690. 22858050 10.1016/j.annepidem.2012.06.105

[20] Subramanian SV, Glymour MM, Kawachi I (2007) Identifying causal ecologic effects on health: a methodological assessment. In: Galea S, editor. Macrosocial Determinants of Population Health. New York, NY: Springer. pp. 301–331.

[21] Suzuki E (2012) Time changes, so do people. Soc Sci Med 75: 452–456. 22591827 10.1016/j.socscimed.2012.03.036

[22] Lakon CM, Godette DC, Hipp JR (2008) Network-based approaches for measuring social capital. In: Kawachi I, Subramanian SV, Kim D, editors. Social Capital and Health. New York, NY: Springer. pp. 63–81.

[23] Luke DA, Harris JK (2007) Network analysis in public health: history, methods, and applications. Annu Rev Public Health 28: 69–93. 17222078 10.1146/annurev.publhealth.28.021406.144132

[24] Rasbash J, Browne WJ, Healy M, Cameron B, Charlton C (2010) MLwiN Version 2.22. Centre for Multilevel Modelling, University of Bristol.

[25] Suzuki E, Takao S, Subramanian SV, Komatsu H, Doi H, et al (2010) Does low workplace social capital have detrimental effect on workers' health? Soc Sci Med 70: 1367–1372. 20176429 10.1016/j.socscimed.2010.01.014

[26] Suzuki E, Fujiwara T, Takao S, Subramanian SV, Yamamoto E, et al. (2010) Multi-level, cross-sectional study of workplace social capital and smoking among Japanese employees. BMC Public Health 10: 489. Available: www.biomedcentral.com/1471-2458/10/489. Accessed 11 October 2012. 10.1186/1471-2458-10-489 PMC2931472 20716334

[27] Schwartz S, Susser E, Susser M (1999) A future for epidemiology? Annu Rev Public Health 20: 15–33. 10352847 10.1146/annurev.publhealth.20.1.15

[28] Susser M, Susser E (1996) Choosing a future for epidemiology: II. From black box to Chinese boxes and eco-epidemiology. Am J Public Health 86: 674–677. 8629718 10.2105/ajph.86.5.674 PMC1380475

[29] Robins G, Snijders T, Wang P, Handcock M, Pattison P (2007) Recent developments in exponential random graph (p*) models for social networks. Social Networks 29: 192–215.

[30] Robins G, Pattison P, Kalish Y, Lusher D (2007) An introduction to exponential random graph (p*) models for social networks. Social Networks 29: 173–191.

[31] Desmarais BA, Cranmer SJ (2012) Statistical inference for valued-edge networks: the generalized exponential random graph model. PLoS One 7: e30136. Available: http://www.plosone.org/article/info%3Adoi%2F10.1371%2Fjournal.pone.0030136. Accessed 11 October 2012. 10.1371/journal.pone.0030136 PMC3261863 22276151

[32] Simpson SL, Hayasaka S, Laurienti PJ (2011) Exponential random graph modeling for complex brain networks. PLoS One 6: e20039. Available: http://www.plosone.org/article/info%3Adoi%2F10.1371%2Fjournal.pone.0020039. Accessed 11 October 2012. 10.1371/journal.pone.0020039 PMC3102079 21647450

[33] Simpson SL, Moussa MN, Laurienti PJ (2012) An exponential random graph modeling approach to creating group-based representative whole-brain connectivity networks. NeuroImage 60: 1117–1126. 22281670 10.1016/j.neuroimage.2012.01.071 PMC3303958

[34] Cummins S, Curtis S, Diez-Roux AV, Macintyre S (2007) Understanding and representing ‘place’ in health research: a relational approach. Soc Sci Med 65: 1825–1838. 17706331 10.1016/j.socscimed.2007.05.036

[35] Diez-Roux AV (2011) Complex systems thinking and current impasses in health disparities research. Am J Public Health 101: 1627–1634. 21778505 10.2105/AJPH.2011.300149 PMC3154209

