# Supplementary material for: Clarifying the Use of Aggregated Exposures in Multilevel Models: Self-Included vs. Self-Excluded Measures
Source: PLoS One. 2012 Dec 10;7(12):e51717. doi: 10.1371/journal.pone.0051717 (PMC3519740; doi:10.1371/journal.pone.0051717)
Supplement: Text S1 — Sample covariance between individual- and group-level social capital variables in models 1, 2a, and 3a. (PDF) [file pone.0051717.s001.pdf]

Clarifying the Use of Aggregated Exposures in Multilevel Models: Self-Included vs. Self-Excluded Measures

Etsuji Suzuki, Eiji Yamamoto, Soshi Takao, Ichiro Kawachi, S. V. Subramanian

Text S1: Sample covariance between individual- and group-level social capital variables in models 1, 2a, and 3a

Unlike the cluster-mean centering procedure (model 2a), the self-exclusion procedure (model 3a) cannot completely evade the collinearity problem encountered in model 1. Below, we compare the sample covariance between individual- and group-level social capital variables in models 1, 2a, and 3a. Symbols are as defined in the main text. In addition, we define the total number of individuals as *N* and the number of groups as *J*. Therefore, the mean of individual-level social capital score for all individuals is $\bar{x} = \sum_{ij} x_{ij} \Big/ N = \sum_{j} n_j \bar{x}_j \Big/ N$.

In model 1 (self-included model without cluster-mean centering), the sample covariance between individual-level social capital (x_{ij}) and group-level social capital ($\bar{x}_j$) is calculated as follows:

$$
\begin{aligned}
S^2_{\text{model 1}} &= \frac{\sum_{ij}\left(x_{ij}-\bar{x}\right)\left(\bar{x}_j-\bar{x}\right)}{N-1} \\
&= \frac{\sum_{ij}\left\{\left(x_{ij}-\bar{x}_j\right)+\left(\bar{x}_j-\bar{x}\right)\right\}\left(\bar{x}_j-\bar{x}\right)}{N-1} \\
&= \frac{\sum_{ij}\left(x_{ij}-\bar{x}_j\right)\left(\bar{x}_j-\bar{x}\right)}{N-1}+\frac{\sum_{ij}\left(\bar{x}_j-\bar{x}\right)^2}{N-1} \\
&= \frac{\sum_{ij}\left(\bar{x}_j-\bar{x}\right)^2}{N-1} \quad \left(\because \sum_{i}\left(x_{ij}-\bar{x}_j\right)=0 \text{ for } \forall j\right) \\
&= \frac{\sum_{j} n_j\left(\bar{x}_j-\bar{x}\right)^2}{N-1} \\
&= S^2_B,
\end{aligned}
$$

which is a *Weighted Between Clusters Variance*. Note that this equation describes the variance of the within-cluster means.

In model 2a (self-included model with cluster-mean centering), since the mean of cluster-mean centered individual-level social capital ($x_{ij} - \bar{x}_j$) is 0, the sample covariance between cluster-mean centered individual-level social capital ($x_{ij} - \bar{x}_j$) and group-level social capital ($\bar{x}_j$) is

$$\begin{aligned} S^2_{\text{model 2a}} &= \frac{\sum_{ij}\left(x_{ij} - \bar{x}_j\right)\left(\bar{x}_j - \bar{x}\right)}{N-1} \\ &= 0. \end{aligned}$$

Thus, as noted previously [1,2], in this model, individual-level social capital variable is orthogonal to its group-level counterpart.

In model 3a (self-excluded model without cluster-mean centering), the sample covariance between individual-level social capital (x_{ij}) and self-excluded group-level social capital ($\bar{x}_{j\backslash i}$) is calculated as

$$\begin{aligned} S^2_{\text{model 3a}} &= \frac{\sum_{ij}\left(x_{ij} - \bar{x}\right)\left(\bar{x}_{j\backslash i} - \bar{x}\right)}{N-1} \\ &= \frac{\sum_{ij}\left\{\left(x_{ij} - \bar{x}_j\right) + \left(\bar{x}_j - \bar{x}\right)\right\}\left\{\left(\bar{x}_j - \bar{x}\right) - \frac{x_{ij} - \bar{x}_j}{n_j - 1}\right\}}{N-1} \quad \left(\because n_j \bar{x}_j = \left(n_j - 1\right)\bar{x}_{j\backslash i} + x_{ij}\right) \\ &= \frac{\sum_{ij}\left(x_{ij} - \bar{x}_j\right)\left(\bar{x}_j - \bar{x}\right)}{N-1} + \frac{\sum_{ij}\left(\bar{x}_j - \bar{x}\right)^2}{N-1} - \sum_{ij}\frac{\left(x_{ij} - \bar{x}_j\right)^2}{\left(N-1\right)\left(n_j - 1\right)} - \sum_{ij}\frac{\left(\bar{x}_j - \bar{x}\right)\left(x_{ij} - \bar{x}_j\right)}{\left(N-1\right)\left(n_j - 1\right)} \\ &= \frac{\sum_{j} n_j\left(\bar{x}_j - \bar{x}\right)^2}{N-1} - \frac{\sum_{j} S^2_{wj}}{N-1} \quad \left(\because \sum_{i}\left(x_{ij} - \bar{x}_j\right) = 0 \text{ for } \forall j\right) \\ &= S^2_B - \frac{J}{N-1}\bar{S}^2_w, \end{aligned}$$

where $S^2_{wj} = \sum_{i}\left(x_{ij} - \bar{x}_j\right)^2 \Big/ \left(n_j - 1\right)$ and $\bar{S}^2_w = \sum_{j} S^2_{wj} \Big/ J$. The former is a *Within Cluster Variance*, and the latter is the *mean of Within Cluster Variances*. Although the self-exclusion procedure (model 3a) can possibly mitigate part of the collinearity problem encountered in model 1, it does not offer a robust solution like model 2a. If cluster size is unvarying, the sample covariance between individual-level social capital (x_{ij}) and self-excluded group-level

social capital ($\bar{x}_{j\backslash i}$) is calculated as follows:

$$S^2_{\text{model 3a}} = \frac{\sum_j n_j \left(\bar{x}_j - \bar{x}\right)^2}{N-1} - \frac{\sum_j S^2_{wj}}{N-1}$$
$$= S^2_B - \frac{N}{N-1}\frac{\sum_j S^2_{wj}}{n\,J}$$
$$= S^2_B - \frac{N}{N-1}\frac{\bar{S}^2_w}{n}$$
$$\approx S^2_B - \frac{\bar{S}^2_w}{n},$$

which describes the variance of the within-cluster means (S^2_B) minus the mean of within-cluster variances divided by cluster size ($\bar{S}^2_w/n$). When the group-level random effect of the intercept is zero, S^2_B is approximately consistent and equal to $\bar{S}^2_w/n$, implying that $S_{\text{model 3a}}$ is also zero. By contrast, when the group-level random effect of the intercept is non-zero, S^2_B increases while $\bar{S}^2_w/n$ does not change. Thus, $S_{\text{model 3a}}$ is expected to increase as the group-level random effect of the intercept increases.

References

1. Raudenbush SW, Bryk AS (2002) Hierarchical Linear Models: Applications and Data Analysis Methods. Thousand Oaks, CA: Sage Publications.
2. Bingenheimer JB, Raudenbush SW (2004) Statistical and substantive inferences in public health: issues in the application of multilevel models. Annu Rev Public Health 25: 53-77.
