# Supplementary material for: Clarifying the Use of Aggregated Exposures in Multilevel Models: Self-Included vs. Self-Excluded Measures
Source: PLoS One. 2012 Dec 10;7(12):e51717. doi: 10.1371/journal.pone.0051717 (PMC3519740; doi:10.1371/journal.pone.0051717)
Supplement: Text S2 — Items used to measure workplace social capital. (PDF) [file pone.0051717.s002.pdf]

Clarifying the Use of Aggregated Exposures in Multilevel Models: Self-Included vs. Self-Excluded Measures

Etsuji Suzuki, Eiji Yamamoto, Soshi Takao, Ichiro Kawachi, S. V. Subramanian

Text S2: Items used to measure workplace social capital

The 20 items used to measure workplace social capital are given below.

1. Trust between co-workers within my work unit is high.
2. Trust between workers and supervisors within my work unit is high.
3. People in my work unit share common perceptions about work.
4. Supervisors and subordinates in my work unit share common perceptions about work.
5. I feel that I genuinely belong to my work unit.
6. I feel that I genuinely belong to this company.
7. People in my work unit cooperate to solve problems.
8. Workers and supervisors in my work unit cooperate to solve problems.
9. It is easy for me to ask favors of my co-workers.
10. If a co-worker in my work unit has personal difficulties, we help him/her.
11. If a co-worker in my work unit has personal difficulties, our supervisor helps him/her.
12. If necessary, people in my work unit are willing to help those in other work units.
13. People in my work unit routinely cooperate with other work units.
14. I find it easy to share my problems with the co-workers in my unit, and to confide in them.
15. I find it easy to share my problems with the supervisors in my unit, and to confide in them.
16. I can rely on the co-workers in my unit if I have a serious problem.
17. I can rely on the supervisors in my unit if I have a serious problem.
18. I frequently socialize with co-workers in my work unit outside of work.
19. Workers and supervisors in my work unit frequently socialize outside of work.
20. I actively participate in club activities in this company.
